# Supplementary material for: Anti-Metatype Antibody Screening, Sandwich Immunoassay Development, and Structural Insights for β-Lactams Based on Penicillin Binding Protein
Source: Molecules. 2021 Sep 13;26(18):5569. doi: 10.3390/molecules26185569 (PMC8470104; doi:10.3390/molecules26185569)
Supplement: Supplementary file 1 [file molecules-26-05569-s001.zip › molecules-1350331-supplementary.pdf]

Supporting Information

Anti- metatype antibody screening, sandwich immunoassay development, and structural insights for β-lactams based on penicillin binding protein

Yuchen Bai, Leina Dou, Weilin Wu, Zhimin Lu, Jiaqian Kou, Jianzhong Shen, Kai Wen*, Zhanhui Wang

College of Veterinary Medicine, China Agricultural University, Beijing Key Laboratory of Detection Technology for Animal Derived Food Safety, Beijing Laboratory for Food Quality and Safety, 100193 Beijing, People's Republic of China

** Author to whom correspondence should be addressed*

Tel: +86–10–6273 4565

Fax: +86–10–6273 1032

E–mail: wenkai@cau.edu.cn

Buffers

The common buffer solutions used in the experiment are listed:

(1) Coating buffer (0.05 mol L^{-1} carbonate buffer, pH 9.6)

(2) Phosphate buffer solution (PBS buffer, 0.01 mol L^{-1}, pH 7.4)

(2) Blocking buffer (2% skim milk powder (w/v))

(3) Washing buffer (PBST, PBS buffer with 0.05% Tween-20 (v/v), pH 7.2)

(4) Boric acid buffer (0.02 mol L^{-1}, pH 9.0, containing 10% DMF)

(5) Stopping reagent (2 mol L^{-1} H_2SO_4)

Table S1 Identification of PBP2x*-drugs.

complex	OD values
PBP2x*-PNG	0.02
PBP2x*-AMP	0.06
PBP2x*-AMX	0.03
PBP2x*-CTX	0.02
PBP2x*-KF	0.05

Table S2. Molecular descriptors of β-lactams.

β-lactams	MW	SA	V_m	E	μ	MPI	PSA	Log P
PNG	334	349.91	383.72	-1421.77	6.56	15.28	227.32	0.84
AZL	461	456.26	506.79	-1889.07	4.38	17.17	307.44	-0.74
AMX	365	365.85	406.47	-1551.59	4.91	15.82	235.05	-0.58
AMP	349	359.57	397.55	-1476.76	5.57	14.89	231.54	-0.2
OXA	402	391.47	448.25	-1665.10	6.46	15.28	256.52	1.5
CLX	437	413.48	469.29	-2123.97	7.50	15.78	282.88	2.06
PIPC	518	513.33	574.90	-2079.76	6.77	17.27	353.25	-0.71
DCX	470	433.68	490.75	-2582.84	6.82	15.18	276.65	2.62
CFM	456	428.85	467.15	-2205.11	4.54	17.45	287.51	0.25
CFN	460	444.25	493.17	-2154.24	18.32	26.67	342.16	-8.29
CPZ	650	608.81	684.48	-2845.33	6.16	18.40	431.69	-0.56
CFAP	423	412.84	455.00	-2060.56	5.74	16.91	277.45	-3.68
CFP	484	462.94	524.20	-2228.60	15.75	25.66	346.09	-5.97
CTRX	558	506.00	562.11	-2843.24	6.15	19.10	349.09	0.02
KF	427	384.85	420.15	-1967.68	12.89	19.55	272.55	0.04
CFT	527	489.19	539.45	-2717.27	4.05	15.86	308.51	1.17
CTX	458	442.07	474.00	-2206.28	4.37	16.41	291.37	0.14
CFZ	463	423.66	46.73	-2465.01	8.86	19.57	308.16	-1.09

molecular weight, MW; van der Waals surface area, SA; molecular volume, Vm; energy, E; molecular dipole moment, μ; molecular polarity index, MPI; polar surface area, PSA; hydrophobic constant, Log P.

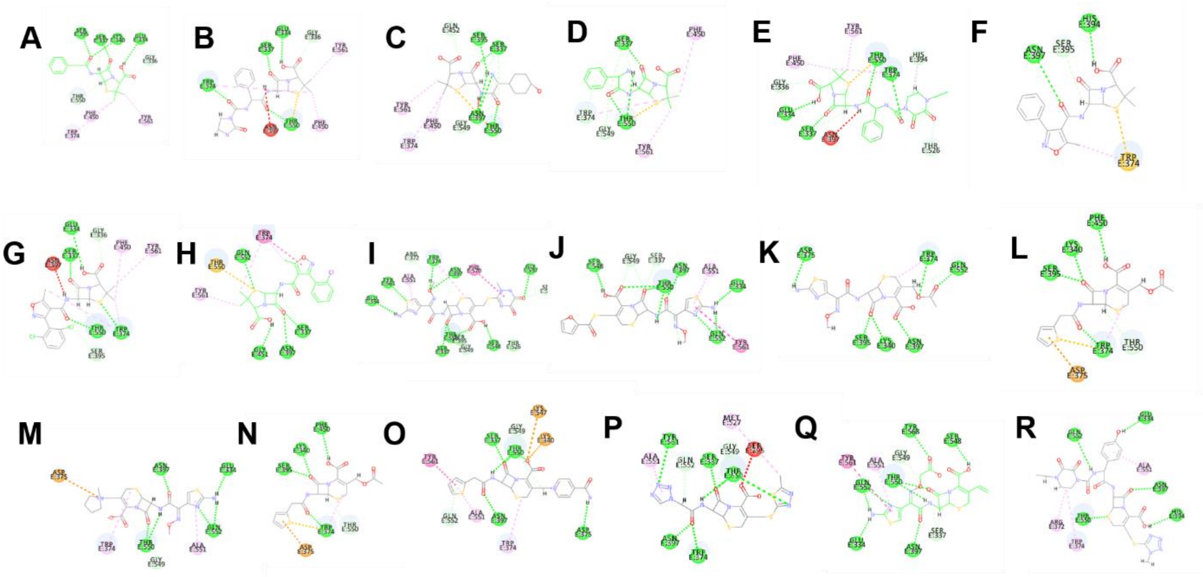

Figure S1. Two-dimensional schematic diagram of the interactions between PBP2x (PDB code: 2z2m) and (A) PNG; (B) AZL; (C) AMX; (D) AMP; (E) PIPC; (F) OXA; (G) DCX; (H) CLX; (I) CTRX; (J) CFT; (K) CTX; (L) CFAP; (M) CFP; (N) KF; (O) CFN; (P) CFZ; (Q) CFM; (R) CPZ. Hydrogen bonds are shown as green dashed lines, hydrophobic interactions are displayed as purple dashes, and the electrostatic force is expressed as orange dashes.
